# Extracellular acidity and ATP modulate ion currents in human cumulus cells indicating possible roles as metabolic sensors of the follicular microenvironment

**DOI:** 10.14814/phy2.70729

**Published:** 2026-01-28

**Authors:** Andrea Biagini, Rosaria Gentile, Cristina Corbucci, Monica Mariani, Alessandro Favilli, Sandro Gerli, Bernard Fioretti

**Affiliations:** ^1^ Department of Medicine and Surgery University of Perugia Perugia Italy; ^2^ Department of Chemistry, Biology and Biotechnologies University of Perugia Perugia Italy; ^3^ Interdepartmental Laboratory of Reproductive Physiopathology University of Perugia Perugia Italy; ^4^ Centre of Assisted Reproductive Technologies S. Maria della Misericordia Hospital Perugia Italy; ^5^ Section of Obstetrics and Gynecology, Department of Medicine and Surgery University of Perugia Perugia Italy

**Keywords:** cumulus cells, female fertility, follicular microenvironment, ion channels, patch clamp, reproductive biology

## Abstract

Cumulus cells (CCs), derived from granulosa cells, play a key role in supporting oocyte maturation and development through bidirectional communication. However, their electrophysiological properties in humans are poorly defined. Here, we characterized ionic currents and their modulation in primary human CCs obtained from patients undergoing in vitro fertilization. Whole cell patch‐clamp recordings identified three electrophysiological sub‐populations: CC‐type 1, expressing voltage‐dependent K^+^ currents supported mainly by potassium voltage‐gated channel subfamily A member 5 (K_V_1.5, KCNA5); CC‐type 2, predominantly showing a barium‐sensitive cationic current attributable to transient receptor potential cation channel subfamily M member 5 (TRPM5); and CC‐type 3, displaying mainly a noisy and voltage‐dependent K^+^ current typical of potassium calcium‐activated channel subfamily M alpha 1 (BK_Ca_, KCNMA1). Pharmacological experiments, immunocytochemistry and rt‐PCR confirmed the molecular expression of KCNA5, TRPM5 and KCNMA1. Mild extracellular acidification (pH = 6.2) rapidly and reversibly blocked TRPM5‐like current, both inward and outward. Furthermore, 100 μM ATP induced metabotropic responses, evoking coupled intracellular Ca^2+^ release and activating TRPM5‐mediated currents, as demonstrated by experiments with patch‐clamp and FURA‐2 calcium imaging. These findings reveal that human CCs integrate extracellular acidity and purinergic signals via distinct ion channels, suggesting a role as electrochemical sensors of the follicular microenvironment.

## Graphical abstract

Extracellular acidity and ATP modify electrophysiological profile in human cumulus cell subpopulations, characterized by differential functional expression of TRPM5‐like, BKCa and K_V_1.5 currents.

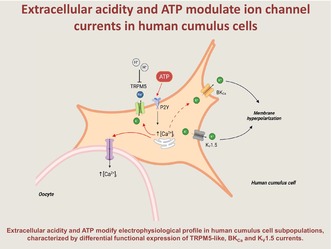

## 1 INTRODUCTION

Cumulus cells (CCs) are somatic cells present in the ovary, which differentiate from granulosa cells (GCs), the main structural elements of the follicle (Mohammed et al., 2019), structurally surrounding the oocyte. During folliculogenesis, in the transition from primordial follicle to primary follicle, GCs undergo differentiation processes that induce morphological changes, passing from a flattened shape during development, through a cuboidal shape during ovulation, to the luteal phase characterized by hypertrophy (Lintern‐Moore & Moore, 1979). These changes are accompanied by structural changes also of the follicle, regulated by paracrine and autocrine growth factors (Skinner, 2005). After antrum formation, GCs differentiate into two different cell populations (Channing et al., 1980), the mural GCs forming the follicle wall with steroidogenic activity and the CCs that surround the oocyte, with which they communicate through cell junctions (Ortiz et al., 1983) and trans‐zonal cytoplasmic projections that insert into the zona pellucida (Albertini et al., 2001), giving rise to a structure defined cumulus‐oocyte complex (COC) (Del Bianco et al., 2024; Gilchrist et al., 2008). Here, a bidirectional communication between the CCs and the oocyte is made possible by the presence of large‐pore channels, the connexins (Cx), which act as intercellular channels (Gilchrist et al., 2008; Russell et al., 2016). Molecules that transit through connexins include growth factors commonly known as oocyte‐secreted growth factors (OSFs), which regulate, e.g., the proliferation of granulosa and CCs and consequently follicular growth (Eppig et al., 2002; Gilchrist et al., 2006), mucification and expansion of CCs, required for ovulation (Dragovic et al., 2005; Ritter et al., 2015; Su et al., 2008; Vanderhyden et al., 1990). Electrophysiological studies in murine COC were conducted and the involvement of purinergic receptors was investigated (Arellano et al., 2002). Patch‐clamp study revealed that mice lacking Cx43 were not electrically coupled and folliculogenesis was impaired (Tong et al., 2006). Among all, Cx43 represents the predominant gap junctions in human CCs (Wang et al., 2009). A morphological classification of the immature COC was also proposed by studying bovine oocytes, and three different populations were observed in relation to the intracellular calcium stores and calcium currents recorded by voltage clamp technique (Boni et al., 2002). L‐type Ca^2+^ currents were found in lambs, and electrical age‐related differences were observed (Boni et al., 2008). However, studies on human CCs are still missing. Therefore, to fill this gap the aim of this study was to characterize electrophysiologically and biophysically the human CCs. The outcomes from this study could help to develop new therapeutic approaches to improve female fertility, targeting ion channels.

## 2 MATERIALS AND METHODS

### 2.1 Cell culture

Human CCs were obtained following oocyte retrieval and decumulation from 28 patients undergoing IVF at the Centre of Assisted Reproductive Technologies, S. Maria della Misericordia Hospital, Perugia, Italy. The study was conducted in accordance with the Declaration of Helsinki and in compliance with the guidelines of the Committee for Publication Ethics (COPE). The study required approval by the local bioethics committee and the Regional Health Authorities (prot. n° NCT05958914). All patients signed an informed consent. All patients involved in the study underwent controlled ovarian stimulation using GnRH antagonist (Orgalutran, Organon, Rome, Italy, ATC code: H01CC01) administration protocols. After collection and decumulation from the oocytes using ICSI Cumulase® (Origio, CooperSurgical, Trumbull, CT, USA, cat. no. 1612) (De Vos et al., 2008; Evison et al., 2009; Taylor et al., 2006), samples from each patient were transported from the hospital to the laboratory at room temperature and immediately processed as described previously (Aghadavod et al., 2015), with modifications. Briefly, samples were transferred into 15 mL tubes (Falcon, Corning, Glendale, AZ, USA) and centrifuged for 10 min at 1100 rpm at 21°C; after each centrifuge, the supernatant was discarded and, if blood was present, 1 mL of Red Blood Cells (RBC) lysis buffer was added. Dulbecco's Phosphate‐Buffered Saline (DPBS, cat. no. ECB4004L) was then added to volume and a new centrifuge was performed. This step was repeated up to 3 times, to eliminate red blood cells. The cell pellet was then suspended in Dulbecco's Modified Eagle Medium high glucose (DMEM, cat. no. ECB7501L) containing 10% fetal bovine serum exosome depleted (FBS, cat. no. ECS8001N), 1% glutamine and 100 U/mL penicillin/streptomycin. Cells were finally plated in 35 mm diameter Petri dishes (Falcon, cat. no. 353001). A variable number of recordings, ranging from one to three, was conducted on cells from each patient. All reagents were purchased from Euroclone (EuroClone S.p.A., Pero, Milan, Italy).

### 2.2 Electrophysiological recordings

Ionic currents in primary human CCs were recorded using the patch‐clamp technique in a whole‐cell dialyzation configuration. The external solution used (modified Ringer, pH = 7.4, titration with NaOH) contained: 140 mM NaCl, 5 mM KCl, 2 mM CaCl_2_, 2 mM MgCl_2_, 5 mM MOPS, 10 mM glucose. The intracellular solution inside the pipette (pH = 7.2, titration with KOH) contained: 155 mM KCl, 1 mM MgCl_2_, 5 mM MOPS, 1 mM EGTA‐K and 30 nM or 1 μM free Ca^2+^ depending on the experiments. Solutions containing different free Ca^2+^ concentrations were prepared by adding an appropriate amount of CaCl_2_ to the stock solution. For the study of currents in the absence of potassium, the intracellular solution (pH = 7.2, titration with CsOH) contained: 115 mM gluconic acid, 30 mM CsCl, 5 mM MgCl_2_, 20 mM HEPES, 10 mM glucose. The blockers used were 5‐(4‐phenoxybutoxy)psoralen (PAP‐1), 4‐aminopyridine (4‐AP, Sigma‐Aldrich, cat. no. A‐0152), 1‐octanol (Sigma‐Aldrich, cat. no. 297887), 5‐nitro‐2‐(3‐phenylpropyl‐amino) benzoic acid (NPPB, Tocris Bioscience, Bio‐Techne, Milan, Italy, cat. no. 0593), tetraethylammonium chloride (TEA, Sigma‐Aldrich, T2265), 5,7‐dimethoxy‐2‐(4‐methoxyphenyl)chromen‐4‐one (trimethylapigenin, TMA, Alomone Labs, Jerusalem, Israel, cat. no. T‐145) and BaCl_2_ (Carlo Erba, Milan, Italy, cat. no. 321757), at concentrations of 10 μM (Gubič et al., 2022; Schmitz et al., 2005), 1 mM (Catacuzzeno et al., 2008), 2 mM (Fioretti et al., 2004), 100 μM (Fioretti et al., 2004), 2 mM (Catacuzzeno et al., 2011), 200 μM and 600 μM, respectively, chosen according to the available literature and to the manufacturer's indications for what concerns TMA (www.alomone.com). Adenosine triphosphate (ATP) was used at a concentration of 100 μM (Sigma‐Aldrich, cat. no. A‐2383) (Fioretti et al., 2005; Fu et al., 2018). All the remaining reagents used were analytical grade and purchased from Sigma‐Aldrich (Merck, Darmstadt, Germany) or Alomone Labs. PAP‐1 was provided by Heike Wulff (University of California, Davis, CA, USA) and Cristina Limatola (University of La Sapienza, Rome, Italy). Glass micropipettes had a resistance of 3–5 MΩ. Currents and voltages were amplified with an EPC‐10 amplifier (HEKA Elektronik GmbH, Reutlingen, Germany) and analyzed with PatchMaster v2x92 (HEKA Elektronik GmbH, Reutlingen, Germany) and Origin Pro 8.5 software (OriginLab Corporation, Northampton, MA, USA). Currents were filtered at 2 kHz and sampled at 40 μs/point. The 2 kHz low‐pass filter was applied to prevent aliasing issues as standard electrophysiology practice, comparing the signal sampling to the filter frequency (Sakmann & Neher, 2013). Membrane capacitance measurements were performed using the PatchMaster transient compensation protocol. Cells that were not compensated properly were discarded from the analysis. Experiments were performed at room temperature (22–25°C).

### 2.3 Calcium imaging

Cells were incubated with 3 μM FURA‐2‐AM (Sigma‐Aldrich, Darmstadt, Germany, cat. no. 47989) for 45 min and washed with Ringer solution (see paragraph above), as previously described (Ragonese et al., 2019). Cells were perfused with ATP 100 μM in continuous use with a gravity perfusion system, focally oriented on the field of interest. The intracellular calcium variation was reported as the change in the ratio between fluorescence emission at 510 nm obtained with excitation wavelengths of 340 and 380 nm (Lambda DG4, Sutter Instruments, Novato, CA, USA). Ratiometric data were acquired every 3 s and fluorescence determinations were performed using the Zeiss AxioExaminer fluorescence microscopy system (Zeiss, Jena, Germany). Healthy cells for imaging were chosen by checking cell morphology (e.g., those with signs of blebbing were discarded) and resting Ca^2+^ level, since necrotic or unhealthy cells usually show a higher resting Ca^2+^ level. At the end of the experiment, 1 μM ionomycin (Tocris Bioscience, Bio‐Techne, Milan, Italy, cat. no. S7074) was applied as a Ca^2+^‐mobilizing agent (Morgan & Jacob, 1994), to exclude cellular death. Acquisition and analysis were performed with ZEN 2 software (Zeiss, Jena, Germany).

### 2.4 rt‐PCR

CC RNA was obtained by TRIzol™ (Invitrogen, Thermo Fisher Scientific, Rodano, Milan, Italy, cat. no. 15596026) after 24 h of cell culture, according to the manufacturer's instructions. Subsequently, cDNA was synthesized by reverse transcription using the QuantiTect® Reverse Transcription Kit (Qiagen, Hilden, Germany, cat. no. 205313). The quality and quantity of RNA and cDNA were assessed by readings on the Infinite M Nano+ spectrophotometer (Tecan, Männedorf, Switzerland). cDNA analysis was performed by Quantinova rt‐PCR with the SYBR® Green PCR Kit (Qiagen, Hilden, Germany, cat. no. 208056) according to the manufacturer's instructions, operated by the Rotor‐Gene Q machine (Qiagen, Hilden, Germany). The target sequences (Invitrogen, Thermo Fisher Scientific, Rodano, Milan, Italy) for this analysis were:

- Peptidylprolyl isomerase A (PPIA). Forward: 5′ TGCTGGACCCAACACAAATG 3′; Reverse: 5′ AACACCACATGCTTGCCATC 3′ (GenBank accession no. NM_021130.3).
- Potassium large conductance calcium‐activated channel (KCNMA1). Forward: 5′ CTAATTCCCAAGGGTTCACAC 3′; Reverse: 5′ GCTTTGCAGAACAGATCACCA 3′ (GenBank accession no. NM_002247).
- Potassium voltage‐gated channel, shaker‐related subfamily, member 5 (KCNA5). Forward: 5′ GGACGAGATACGCTTCTACC 3′; Reverse: 5′ AGATGAGGATAACCAAGACCGAG 3′ (GenBank accession no. N/A).
- Transient receptor potential cation channel subfamily M member 5 (TRPM5). Forward: 5′ ACAGATCAACTACTGCTCGGTGCT 3′; Reverse: 5′ TGTTCCCAGCCATCTAAACCACCT 3′ (GenBank accession no. NM_014555).

The reference gene peptidylprolyl isomerase A (PPIA) was used as an internal control to verify the results of rt‐PCR, showing no changes in expression in the samples studied. The rt‐PCR reaction included 100 ng cDNA, 100 nM forward and reverse primers (Thermo Fisher Scientific, Rodano, Milan, Italy), 10 μL SYBR Green PCR Master Mix (Qiagen, Hilden, Germany) and RNA‐free water up to a volume of 20 μL. The reaction conditions were as follows: 2 min at 95°C for an initial denaturation and enzyme activation, followed by 38 cycles with denaturation at 95°C for 5 s and annealing/elongation at 56°C for 10 s. Negative controls (NTC) were performed with RNA‐free water in place of cDNA. All samples were amplified in duplicate. The specificity of the amplification was tested at the end of the rt‐PCR by melting curve analysis. Gene expression levels were calculated by the ΔCT method, as described by Schmittgen and Livak (2008), normalized by geometric mean of the levels of the reference gene (Cadenas et al., 2022).

### 2.5 Immunocytochemistry

Immunocytochemistry on CCs was performed as described by (Lee et al., 2012), with modifications. CCs cultured on round cover slips coated with poly‐L‐lysine (LaBoindustria S.p.A, Arzergrande, Padua, Italy, cat. no. A1018) were fixed with 4% paraformaldehyde in 0.1 M phosphate‐buffered saline (PBS, BioWhittaker, Lonza, Basil, Switzerland, cat. no. BE17‐516F) for 30 min, washed three times with PBS and pre‐incubated in blocking buffer for 2 h at room temperature. Cells were then washed and incubated at 4°C overnight with primary rabbit polyclonal antibodies anti‐KCNMA1 (ThermoFisher, cat. no. PA1‐923), anti‐TRPM5 (Alomone Labs, cat. no. ACC‐045) or anti‐KCNA5 FITC‐conjugated (Alomone Labs, cat. no. APC‐150‐F), diluted 1:100 in PBS. After washing, cells were incubated in dark at room temperature for 1 h 30 min with Alexa Fluor 555‐conjugated goat anti‐rabbit IgG (ThermoFisher, cat. no. A21428) diluted 1:500 in PBS, to detect KCNMA1 or TRPM5 expressions. Nuclei were stained by applying a 4′,6‐diamidino‐2‐phenylindole (DAPI) solution (Sigma‐Aldrich, cat. no. MBD0015) diluted 1:1000 in PBS. Stained cells were wet‐mounted on glass slides and images were acquired using a fluorescent microscopy AxioExaminer (Zeiss, Jena, Germany).

### 2.6 Statistical analysis

All results are expressed as mean ± standard error (SE), if not specified, in order to compare group means and evaluate the precision of those mean estimations. Differences were evaluated by *t*‐test (two‐sample or pair‐sample), one‐way ANOVA, and chi‐squared test, depending on the necessity. Post‐hoc corrections were performed by using the Holm test and Tukey's HSD, when necessary. *p* < 0.05 was considered statistically significant. Statistical analysis was performed with Origin 8.5 software.

## 3 RESULTS

### 3.1 Electrophysiological characterization

**FIGURE 1:**
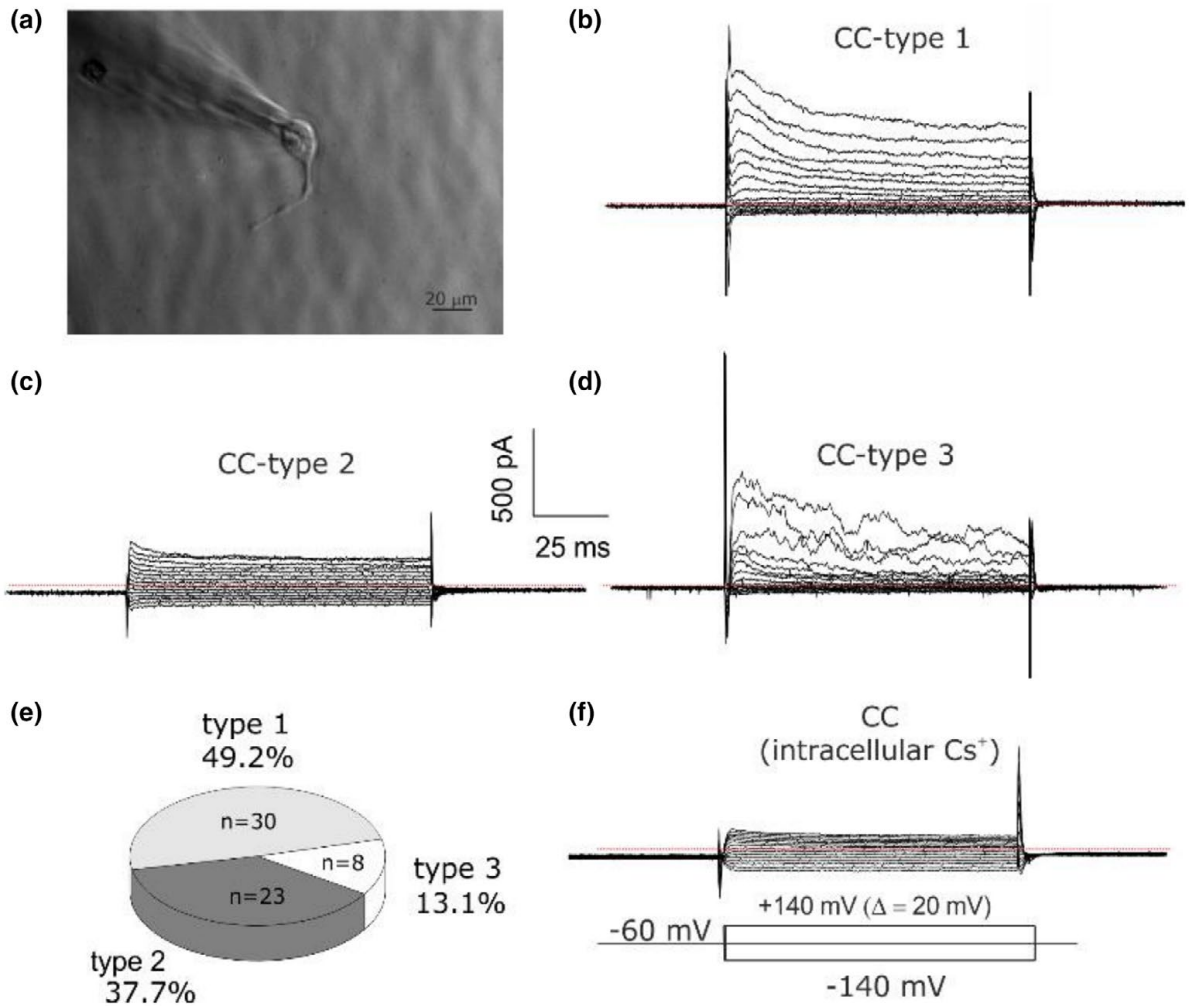
Electrophysiological sub‐populations in human cumulus cells. (a) Cumulus cells, characterized by the presence of filopodia, spindle‐like ectoplasmic projections, were analyzed with the patch‐clamp technique after 24 h of seeding. (b–d) Currents family recording obtained by application of voltage steps from −140 to +140 mV with increments of 20 mV lasting 100 ms (V holding = −60 mV), highlighted the presence of voltage‐dependent currents with outward rectification (b, CC‐type 1), of linear or ohmic currents (c, CC‐type 2) and of voltage‐dependent currents with strong noise (d, CC‐type 3). The red dashed line represents *I* = 0 pA. (e) Pie chart of the frequency of observation of cumulus cells sub‐populations. (f) Currents family recording obtained with intracellular potassium replaced by cesium (*n* = 7, *p* < 0.01, chi‐squared test, three independent experiments). In the bottom the protocols used to obtain the currents family in panel (b–d) and (f).

All CCs analyzed after 24 h of culture presented cellular extensions (Figure 1a), ascribable to filopodia or lamellipodia (Kennedy et al., 1994). Electrophysiological recordings were performed using the patch‐clamp technique in whole‐cell dialyzed configuration. The application of voltage steps from −140 to +140 mV with an increase of 20 mV lasting 100 ms (V holding = −60 mV) highlighted primarily the presence of a voltage dependence (outward rectification) in the majority of the studied cells (Figure 1b,d,e), whereas the others were characterized by non‐voltage‐dependent (linear) currents (Figure 1c,e). Specifically, the first set of experiments was conducted on 61 CCs analyzed from 15 different patients (age 36.1 ± 5.6 years). 49.2% of the analyzed cells (30/61) showed a prominent outward rectification with a behavior typical of voltage‐dependent currents partially inactivated during the voltage depolarization steps (Figure 1b). This population has been defined as “cumulus cell type 1” (CC‐type 1) because of its predominance over the others. A second type of sub‐population was characterized by the absence of voltage‐dependent behaviors, showing a linear or ohmic behavior (Figure 1c). This type of cell was observed with a frequency of 37.7% (23/61) and has been defined as “CC‐type 2”. Finally, less frequently (8/61), a sub‐population of cells with an outward rectifying current characterized by outward rectification with strong noise currents (Figure 1d), different from the “CC‐type 1”, and for this reason called “CC‐type 3”. No significant differences were found in cell capacitances within the three sub‐populations with 18.1 ± 1.7, 20.6 ± 1.9 and 22.3 ± 3.0 pF for type 1, 2 and 3 respectively. A comparative study between the sub‐populations was also performed by evaluating the currents measured at the peak and after 100 ms from the application of the step. The mean peak currents at the positive potential of +140 mV of CC‐type 1, CC‐type 2 and CC‐type 3 were 603 ± 47 pA (*n* = 26), 442 ± 37 pA (*n* = 23), 1052 ± 288 pA (*n* = 8), respectively, and CC‐type 3 was significantly higher than CC‐type 1 and CC‐type 2 (one‐way ANOVA *p* = 0.0005). After intracellular potassium ions replacement with cesium ions, no electrophysiological profile of CC‐type 1 and CC‐type 3 populations was observed in 7 cellular recordings, while in all the recordings, the currents were similar to those ascribed to the CC‐type 2 population (Figure 1f, *n* = 7, *p* < 0.01, chi‐squared test). These data indicate that (1) the outward currents of CC‐type 1 and CC‐type 3 are voltage‐dependent potassium currents; (2) an ubiquitous, nonspecific cation current is present in all cells, mainly expressed in CC‐type 2.

**FIGURE 2:**
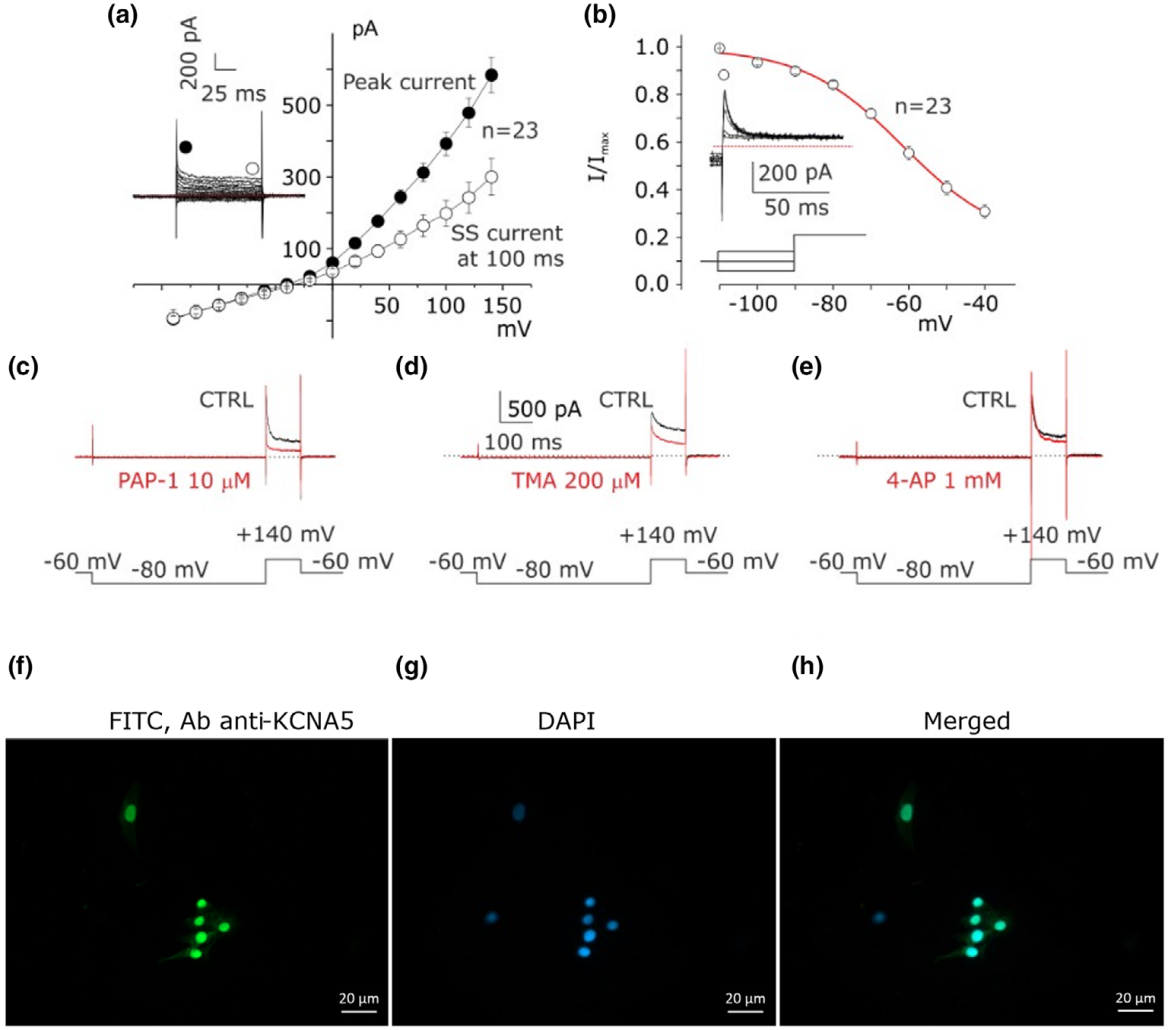
CC‐type 1 expresses Kv1.5 potassium currents. (a) I–V relationship between the peak current and at 100 ms (*n* = 23, seven independent experiments). Inset: Representative current traces family used to build the I–V relationship by using the protocol described in Figure 1. The red dashed line represents *I* = 0 pA. (b) The voltage‐dependent inactivation was studied by plotting the peak of the test current as a function of the pre‐conditioning potential, interpolating the values obtained according to the Boltzmann equation (Equation 1), with $V_{1/2}$ = −55.7 mV and *k* = 13.9 (*n* = 23, seven independent experiments). Inset: Representative steady state current inactivation obtained by conditioning the cell from −110 to −40 mV with an incremental test pulse of 20 mV. (c) Sensitivity of potassium currents before (CTRL, black trace) and after (red traces) application of 10 μM PAP‐1 (three independent experiments). (d) Sensitivity of potassium currents before (CTRL, black trace) and after (red traces) application of 200 μM TMA (three independent experiments). (e) Sensitivity of potassium currents before (CTRL, black trace) and after (red traces) application of 1 mM 4‐AP (three independent experiments). Inset (c–e): Protocol used to study pharmacology profile by pre‐conditioning the cell at −80 mV for 500 ms, then bringing it to +140 mV from a V holding potential (−60 mV). (f) Fluorescence imaging by immunocytochemistry revealed the expression and localization of K_V_1.5 channels in cumulus cells (three independent experiments). K_V_1.5 channel expression was obtained by using primary rabbit IgG polyclonal antibody FITC‐conjugated (1:100). (g) Nuclei were stained with DAPI (1:1000). (h) Merged with Ab anti‐KCNA5‐FITC conjugated and DAPI is displayed. Scale bar represents 20 μm.

The biophysical and pharmacological properties of potassium currents expressed in CC‐type 1 were investigated. The mean I–V relationship built by plotting the peak currents (inset Figure 2a, black circle) and after about 100 ms (inset Figure 2a, empty circle) is displayed in Figure 2a where a clear grade of inactivation was observed. In 30 cells the steady state of inactivation on the K^+^ current of CC‐type 1 was studied by using conditioning protocols from −110 to −40 mV (incremental steps of +10 mV) followed by a test pulse at +20 mV (inset in Figure 2b). The mean peak currents normalized to peak maximal current evocated by conditional step at −110 mV in function to conditional steps was fitted according to the Boltzmann equation (red line in Figure 2b):

(1) $$\frac{I}{I_{\max}}=\frac{G}{\left(1+e^{\left(V-V_{1/2}\right)/k}\right)}+C$$

where *G* and *C* represent the normalized inactivation and constant fractions, respectively, the $V_{1/2}$ represents the voltage where the $\frac{I}{I_{\max}}=0.5$ and *k* the slope factor. In contrast, no inactivation process was observed at 100 ms after the end of the test pulse (see inset in Figure 2b). These data suggest that the potassium currents are a combination of transient potassium currents ($I_{A}$, evaluating meanly the peak current) and a non‐inactivating potassium current component that displays similar features to the previously reported potassium currents ultrarapid activated non‐inactivating ($I_{\mathrm{Kur}}$, evaluated at 100 ms of test pulse).

In order to identify the K^+^ currents in CC‐type 1, pharmacological studies were conducted using blockers PAP‐1 (Gubič et al., 2022; Schmitz et al., 2005), TMA (Liu et al., 2012) and low concentration of 4‐AP (Catacuzzeno et al., 2008). The peak current was sensitive to 10 μM PAP‐1 application but insensitive to 200 μM TMA and 1 mM 4‐AP, whereas the non‐inactivating component (at 100 ms) was sensitive to all blockers (Figure 2c–e). Altogether, pharmacological and biophysical data indicate that the $I_{\mathrm{Kur}}$ current was supported by the K_V_1.5 channel subunit. Immunocytochemistry (Figure 2d–f, Figure S3A) and transcriptomic analyses using rt‐PCR (*n* = 7, Figure S4) confirmed the gene expression of *KCNA5*, encoding for the K_V_1.5 channel subunit. The molecular nature of the I_A_ was not further investigated.

**FIGURE 3:**
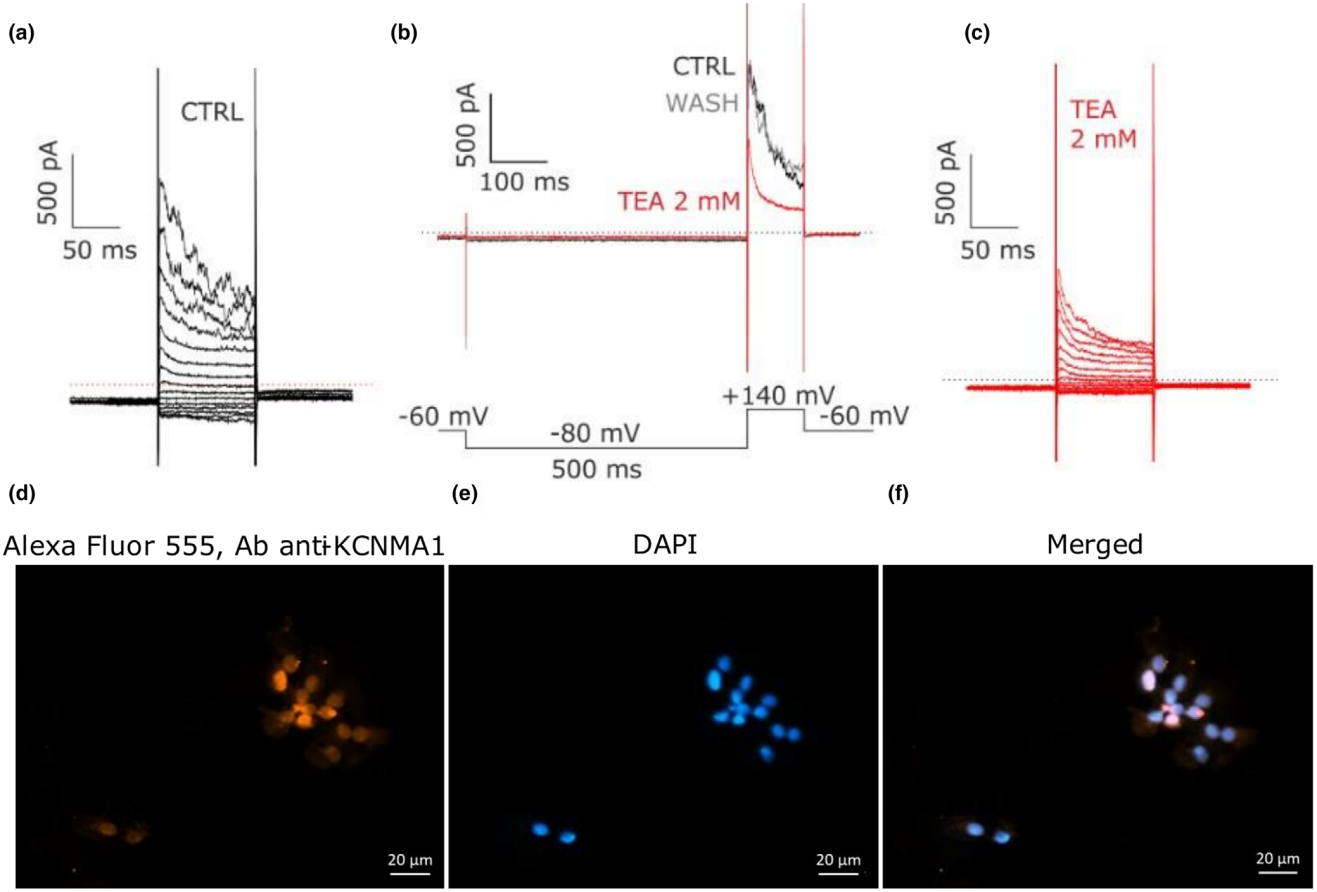
CC‐type 3 expresses BK_Ca_ potassium currents. (a) Recording of a noisy outward rectifying potassium current. The red dashed line represents *I* = 0 pA. (b) Pharmacological steps were performed by pre‐conditioning the cell at −80 mV for 500 ms, then bringing it to +140 mV from V holding potential (−60 mV). The external application in perfusion with 2 mM TEA determined the reversible inhibition of the voltage‐dependent K^+^ current, with removal of the noise (*n* = 8, 5 independent experiments). The black trace represents the current before the application of 2 mM TEA (red trace), whereas the gray trace is the current after the wash. (c) Recording of the TEA‐resistant potassium current after 2 mM TEA applicationon the same cell displayed in (a) and (b) by using the protocol described in Figure 1. (d) Fluorescence imaging by immunocytochemistry revealed the expression and localization of BK_Ca_ channels in cumulus cells (three independent experiments). BK_Ca_ channel expression was obtained by using primary rabbit IgG polyclonal antibody (1:100) and Alexa Fluor 555‐conjugated goat anti‐rabbit (1:500). (e) Nuclei were stained with DAPI (1:1000). (f) Merged with Ab anti‐KCNMA1 (Alexa Fluor 555) and DAPI is displayed. Scale bar represents 20 μm.

In CC‐type 3 the outward rectifying K^+^ current is blocked by 2 mM TEA in a rapid, complete and reversible manner (*n* = 5, Figure 3a–c): the sensitivity to TEA (Catacuzzeno et al., 2011), the voltage‐dependence and the noisiness strongly indicates that this current flows through large‐conductance calcium‐activated potassium channels (BK_Ca_). *KCNMA1* gene expression, encoding for BK_Ca_, was confirmed by rt‐PCR (*n* = 7, Figure S4) and immunocytochemistry (Figure 3d–f, Figure S3B), which revealed a predominant presence near the nuclei and a weak localization in the membrane. Based on the poor frequency of this current, we did not proceed with further investigations. The resting membrane potential measured by zeroed currents in I‐V relationship display that CC‐type 1 was more negative than type 2 and 3 whereas any difference was found between type 2 and 3 (ANOVA test = 0.003; −44.5±3.0, −31.1±2.8 and −29.2±6.2 mV in CC‐type1 (*n* = 29), 2 (*n* = 23) and 3 (*n* = 8) respectively).

**FIGURE 4:**
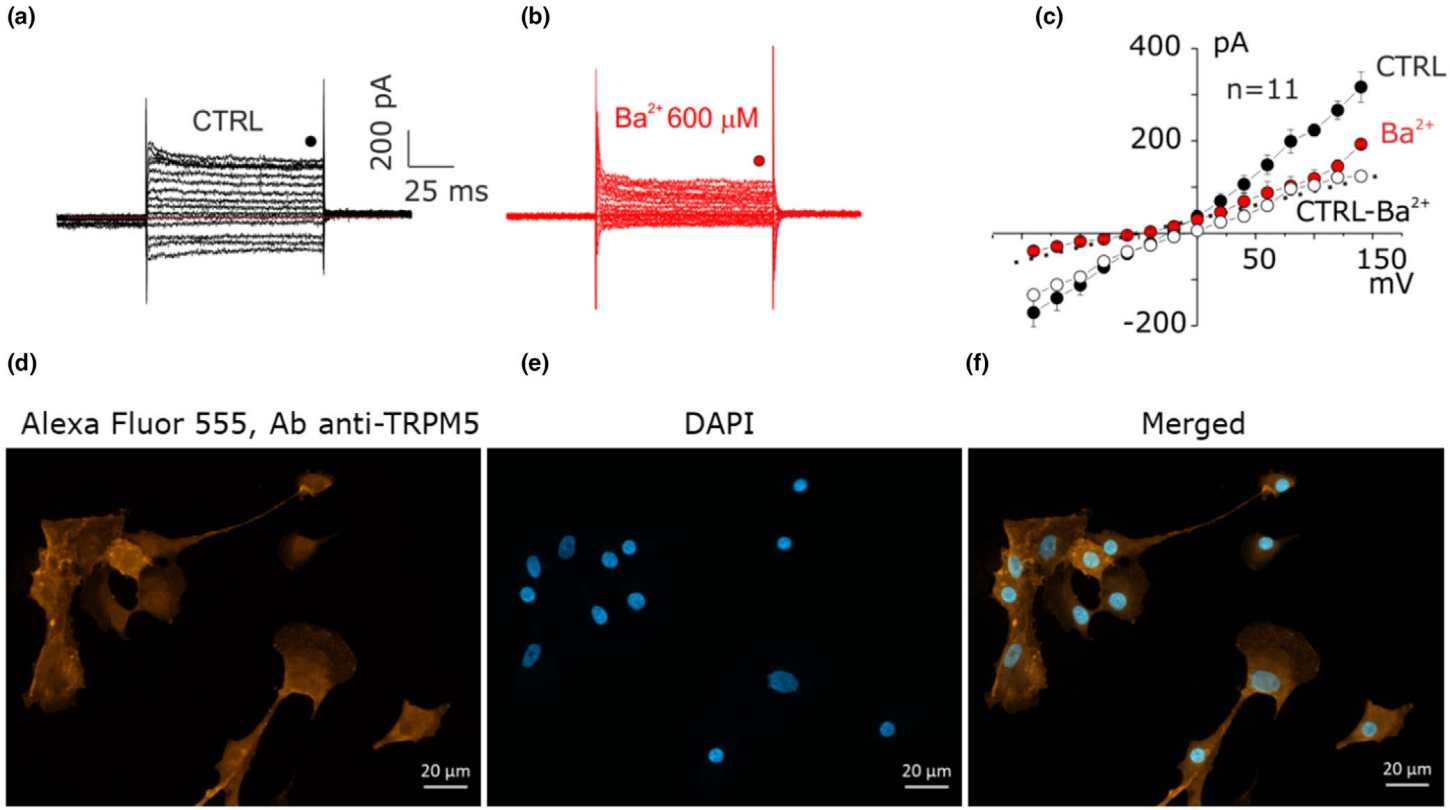
CC‐type 2 expresses TRPM5 cationic currents. (a) Recording before the application of 600 μM Ba^2+^. The red dashed line represents *I* = 0 pA. (b) The application of Ba^2+^ at a concentration of 600 μM determined the irreversible inhibition of the nonspecific cationic current both inwardly and outwardly (*n* = 11, *p* = 0.02, four independent experiments). The protocol using in (a) and (b) are described in Figure 1. (c) The I–V diagram shows the comparison of the currents as a function of the voltage obtained before (black dots) and after (red dots) the application of 600 μM Ba^2+^, while the white dots represent the current that remains by subtracting the current inhibited by Ba^2+^ from the initial one. (d) Fluorescence imaging by immunocytochemistry revealed the expression and localization of TRPM5 channels in cumulus cells (three independent experiments). TRPM5 channel expression was obtained by using primary rabbit IgG antibody (1:100) and Alexa Fluor 555‐conjugated goat anti‐rabbit (1:500). (e) Nuclei were stained with DAPI (1:1000). (f) Merged with Ab anti‐TRPM5 (Alexa Fluor 555) and DAPI is displayed. Scale bar represents 20 μm.

Finally, the current expressed in the CCs‐type 2 population was studied. 600 μM Ba^2+^ irreversibly blocked the current (*n* = 11, mean currents at −140 mV were −209 ± 35 and −73 ± 14 pA in CTRL and 600 μM Ba^2+^, respectively, *p* = 0.02, Figure 4a,b). The blocked current (white dots, Figure 4c) shows a reversal potential close to 0 mV, indicating the nonselective cationic nature. In contrast, this current was insensitive to 2 mM octanol (*n* = 3, Figure S1a) and 100 μM NPPB (*n* = 4, Figure S1b), which were used to exclude the potential participation of connexins and volume‐activated chloride channels, respectively (Fioretti et al., 2004). Since the nonselective cationic current is basically active, we performed FURA‐2 calcium imaging to evaluate calcium permeability modifying Ringer solution with 5 mM of Ca^2+^ or 1 mM Ca^2+^. The extracellular calcium changes did not modify intracellular calcium levels (Figure S2), indicating that cationic current was calcium‐impermeable. These results suggest the involvement of TRPM5 channel based on calcium permeation, transcriptomic analysis by rt‐PCR (*n* = 7, Figure S4) and immunocytochemistry (Figure 4d–f, Figure S3C), confirming the hypothesis accordingly to previous works (Semplici et al., 2021).

### 3.2 Metabolic response of CCs

**FIGURE 5:**
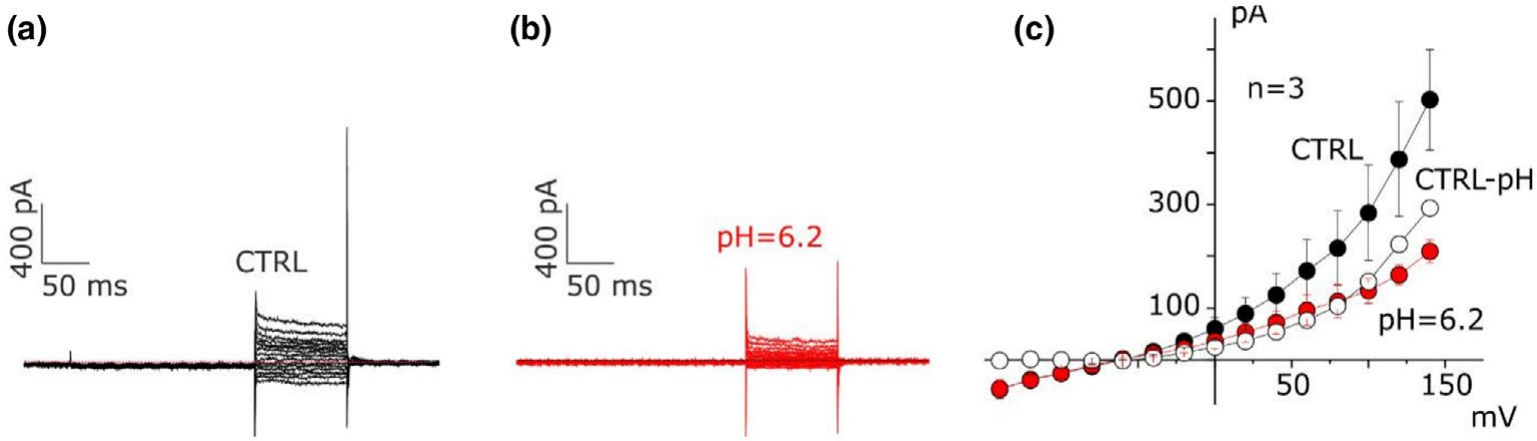
Mild acidification of the extracellular solution inhibits TRPM5 currents in CC cells. (a, b) Recording before (a) and after (b) external acidification (pH = 6.2) in a typical CC‐type 2. (c) The mean I–V diagram shows the comparison of the currents as a function of the voltage obtained before (black dots) and after (red dots) the application of extracellular Ringer solution (pH = 6.2), while the white dots represent the current that remains by subtracting the current inhibited protons from the initial one (*n* = 3, *p* = 0.04, three independent experiments).

After characterizing the ionic nature of the currents, the focus moved to the study of the functional role of these channels. Follicular fluid represents a specific milieu rich in chemical messengers and metabolites, essential for the correct development of the oocyte (Del Bianco et al., 2024). In this context, the anaerobic metabolism produces lactate in the last phases of folliculogenesis, whereas ATP is commonly exchanged within the follicle (Del Bianco et al., 2024). Therefore, we aimed to verify the effect of extracellular acidification and ATP application on ionic currents. Interestingly, we observed in CC‐type 2 (characterized mainly by TRPM5‐like currents) the rapid and reversible block of the cation current induced by mild acidification of the extracellular solution (pH = 6.2, *n* = 3, mean currents at +140 mV were 503 ± 97 and 209 ± 22 pA in CTRL and pH = 6.2, respectively, *p* = 0.04; Figure 5a–c) (Liu et al., 2005).

**FIGURE 6:**
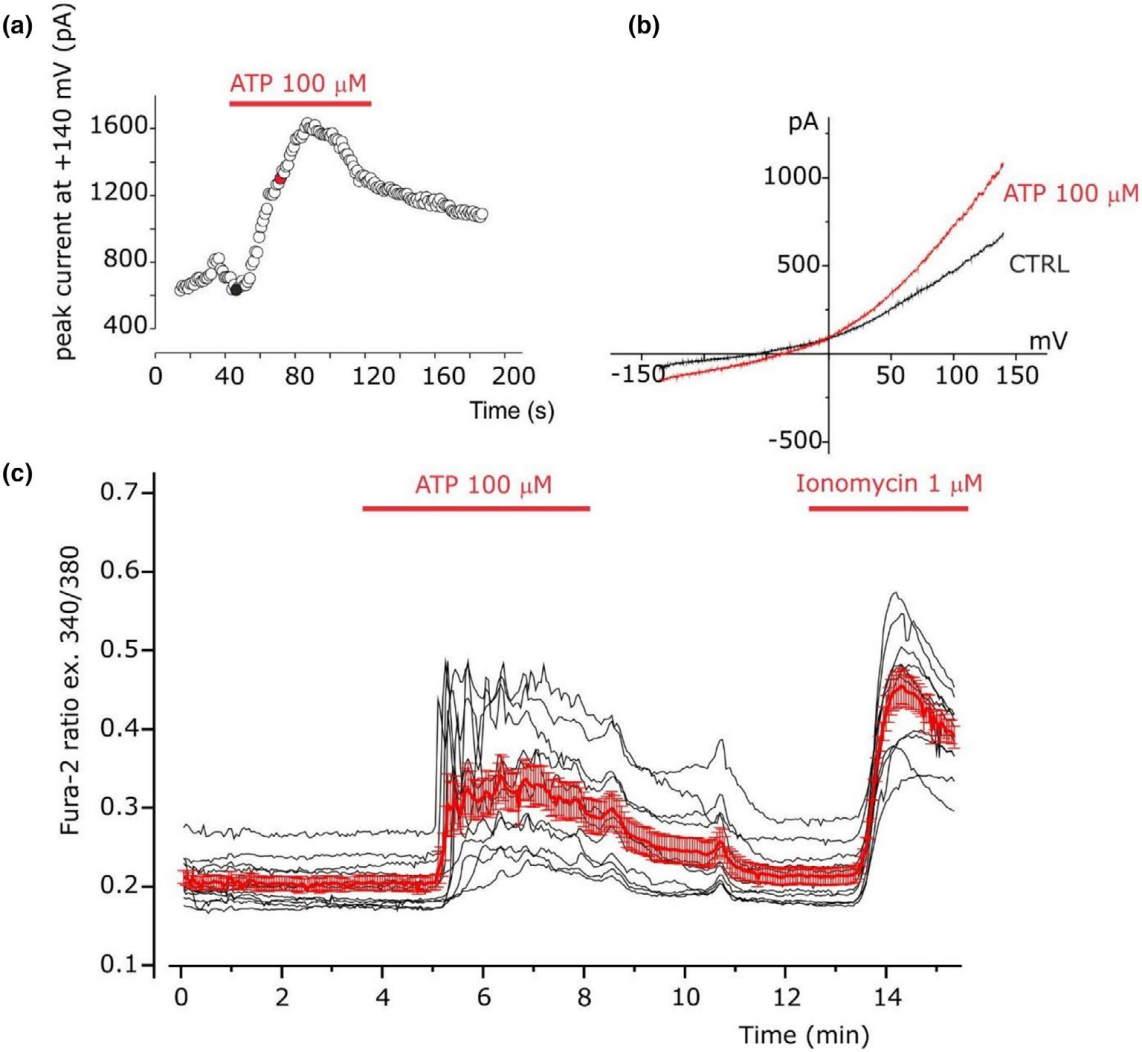
Extracellular ATP increases intracellular calcium and cation currents with similar temporal profiles. (a) Time course of the peak current at +140 mV during the application of 100 μM ATP in the extracellular solution. (b) Example of the current recorded before and after the application of 100 μM ATP in the extracellular solution by applying linear voltage ramp protocol of 1.1 s from −140 mV to +140 (Vh = −60 mV). (c) Time course of the calcium imaging in cumulus cells (*n* = 14, *p* = 0.00006, three independent experiments) loaded with FURA‐2 showing the changing of the fluorescence ratio 340/380 in human cumulus cells during the application of 100 μM ATP in the extracellular solution and 1 μM ionomycin. The bold red line represents the average fluorescence ratio 340/380 with the error bar (SE) of the single traces (black line) corresponding to each cell analyzed.

Finally, we tested the effect of the purinergic signal by extracellular perfusion of 100 μM ATP (Fu et al., 2018). The super‐infusion of ATP during electrophysiological recording in perforated whole cell configuration induced a slow increase of the outward current at +140 mV. The increase needed few minutes and was partially reversible according to the metabotropic transduction signal promoted by purinergic receptor (Figure 6a). The I–V relationship built with the current recorded before the 100 μM ATP application and at the peak showed that ATP promotes the activation of a nonselective cationic current (Figure 6b), as indicated by the reversal potential close to 0 mV (equilibrium potential of potassium and sodium ions, calculated by Nernst's equation, in our experimental condition was about −90 and + 65 mV, respectively). Since electrophysiological recordings and calcium imaging measure different aspects of cellular activity, with electrophysiology detecting the influx of calcium ions across the plasma membrane, and calcium imaging measuring the resulting accumulation of calcium within the cytosol, in a different cell batch of CCs we performed calcium imaging recordings by using FURA‐2 probe. Calcium imaging recordings displayed a significant increase and oscillatory waves of the intracellular calcium levels after 100 μM ATP application with a similar temporal profile of nonselective cationic current activation (compare Figure 6a with Figure 6c) accordingly with calcium‐dependent activation of TRPM5 channel (*n* = 14, *p* = 0.00006; Figure 6c) (Prawitt et al., 2003).

## 4 DISCUSSION

In this study, the electrophysiology of the CCs was investigated, due to the close communication with the oocyte which can affect the oocyte quality (von Mengden et al., 2020), through a bidirectional communication mediated by gap junctions, i.e., connexins (Wang et al., 2009). The analysis of CCs gene expression has revealed a direct relationship between genes expressed in CCs and the success of fertilization of the corresponding oocyte (Wathlet et al., 2012). In vitro culture of CCs with fetal bovine serum and physiological and supra‐physiological follicle‐stimulating hormone (FSH) concentrations also showed the ability of these cells to synthesize estradiol, thus making its addition unnecessary in the culture media used for the in vitro maturation of immature human oocytes collected from patients undergoing cycles of stimulation with gonadotropins (Chian et al., 1999). As already observed in the literature (Chermuła et al., 2020), after 24 h of in vitro culture the analyzed cells presented a rounded and upright, or star‐shaped, phenotype due to the presence of filopodia or lamellipodia (Kennedy et al., 1994), induced by FSH (Eppig, 1979) and precursors of transzonal projections (TZP), which play a key role in oocyte development (Crozet et al., 2023). After 24 h, CCs change their shape towards a flattened phenotype, accompanied by an increase in the extracellular matrix and collagen production (Clarke, 2022; Kennedy et al., 1994).

Here, three types of currents were characterized biophysically and pharmacologically (Figure 1): (1) a voltage‐dependent inactivating potassium current (I_A_) plus an ultrarapid activating non‐inactivating potassium current sensitive (I_Kur_) to 10 μM PAP‐1 and 200 μM trimethylapigenin, (2) a nonspecific cationic current (TRP‐like) blocked by barium and extracellular acidity and activated by 100 μM ATP, (3) a large‐conductance potassium current (BK_Ca_) sensitive to low concentrations of TEA. Three cellular sub‐populations were then defined based on the expression of the currents just described: CC‐type 1 composed of TRP‐like, I_A_, and I_Kur_ currents (frequency of 49.2%), CC‐type 2 composed predominantly of TRP‐like currents (frequency of 37.7%), and CC‐type 3 presenting all three currents (frequency of 13.1%).

### 4.1 Voltage‐dependent potassium currents in human CCs

Potassium channels are ubiquitously distributed throughout various types of cells, where they control several physiological processes, including volume regulation, migration, proliferation, angiogenesis, and cell apoptosis (Comes et al., 2015). However, the role of potassium channels in the reproductive system is still unclear. To date, an ultrarapid‐activating, slow‐inactivating current (I_Kur_), attributable to K_V_1.x channels, has been identified in mammalian GCs (Kusaka et al., 1993; Mason et al., 2002; Mattioli et al., 1993). Potassium channels belonging to the K_V_1.x family have already been observed in both porcine (Li et al., 2003; Mason et al., 2002) and in humans (Ragonese et al., 2021), where inhibition with 4‐AP reduced the release of progesterone from GCs, and resveratrol reduced the functional expression of an I_Kur_ current associated with plasma membrane depolarization, promoting the increase of intracellular Ca^2+^ (Ragonese et al., 2021), respectively. I_A_ current associated with KCND2 (K_V_4.2) channels has been observed in human GCs, inhibited at physiological concentrations by progesterone, estradiol, and testosterone, and therefore associated with steroidogenesis processes (Kunz et al., 2006). In our experiments, 4‐AP was not effective on the current, but a concentration of 1 mM was adopted, different from the study reported, in which a concentration of 5 mM was used in perfusion, or 2 mM in chronic for 24 and 48 h (Kunz et al., 2006; Li et al., 2003). Biophysical (inactivation) and pharmacological properties of I_K_ in CC‐type 1 confirmed the presence of a voltage‐dependent current. According to our findings, CC‐type 1 is characterized by the presence of a partially inactivating, voltage‐dependent potassium current complex. Biophysical and pharmacological investigations suggest the presence of a rapidly inactivating component ($I_{A}$) and a rapidly activating, non‐inactivating component (*I*_Kur_). The molecular identification of these currents is complex: *I*_A_ is likely mediated by K_V_4.2 and K_V_4.3 subunits (K_V_1.4 was excluded based on insensibility to PAP‐1), whereas I_Kur_ is associated with K_V_1.x subunits, most likely K_V_1.5 (Fedida et al., 1999; Lagrutta et al., 2006), which is blocked by 200 μM TMA, a synthetic selective blocker for voltage‐gated potassium channels KCNA5 (K_V_1.5), and by 10 μM PAP‐1 (Figure 2c–e). This channel is involved in cell cycle regulation, nitric oxide production, and control of resting membrane potential (Wettwer & Terlau, 2014). K_V_1.5 supports an ultrarapid delayed rectifying potassium current activation and a slow inactivation, which is in line with our findings. However, further studies are needed to clarify the involvement of other voltage‐dependent potassium currents in CCs.

Large‐conductance calcium‐activated potassium channels (BK_Ca_) have already been identified in human GCs (Kunz et al., 2002; Traut et al., 2009). Observing the recordings, the removal of noise by 2 mM TEA reveals the coexistence of BK_Ca_ with K_V_1.5 channel currents in CC‐type 3 (Figure 3a–c), suggesting functional modifications of these cells over time, with the appearance of different channels that could also be correlated to the phase of the menstrual cycle of the patient (Zhang et al., 2012) or cell‐cycle dependent (Chubinskiy‐Nadezhdin et al., 2019). In our study, we did not proceed further with the investigation due to the poor presence of the channel within the population; however, we do not exclude that in the future we will analyze its role together with TRPM5, since the rise of intracellular calcium induced by purinergic signaling pathway may activate BK_Ca_ channel and hyperpolarize the membrane.

### 4.2 Nonselective cationic currents in human CCs

TRPM5‐like current identified in this study appears ubiquitous, but associated predominantly with the CC‐type 2 electrophysiological phenotype, blocked by 600 μM Ba^2+^ non‐reversibly (Figure 4a–c) and reversibly by protons (pH = 6.2, Figure 5), activated after the external application of ATP 100 μM (Figure 6), followed by the onset of intracellular calcium increase and oscillations and the opening of the ion‐conducting pore (Prawitt et al., 2003): this confirms the hypothesis previously presented regarding the existence of not a single population, but rather of multiple populations even in the same plate, probably in the transition phase from one maturation state to another. TRP channels are activated by several stimuli including intra‐ and extracellular messengers, chemical, mechanical and osmotic stress, and in relation to intracellular calcium concentration (Clapham, 2003). TRP channels, in particular TRP channel polycistin 1–2 (TRPP1‐2, PKD1‐2) and TRP channel subfamily V member 4 (TRPV4), have been found in murine ovarian and oviduct tissue, expressed mainly in GCs of antral follicles and in correspondence with sensory cilia in the oviduct (Teilmann et al., 2005); their presence, moreover, was increased following stimulation with gonadotropins. The expression of TRPP2 has also been highlighted in the rat corpus luteum (Obermüller et al., 1999). This suggests a functional role of TRP channels, especially TRPP1‐2, as sensors for the regulation of GC differentiation, but also in the development and maturation of the ovarian follicle in relation to changes in the surrounding microenvironment such as the temperature of the follicular fluid or osmotic pressure, which are then transformed into calcium influxes (De Clercq & Vriens, 2018).

Combining electrophysiological and biophysical studies, all the clues let us hypothesize that the recorded current was supported by TRPM5, since this channel is the only one, together with TRPM4, to carry just monovalent cations. However, despite being Ca^2+^‐impermeable, TRPM5 is rapidly activated by intracellular rise of Ca^2+^ up to 1 nM (Prawitt et al., 2003), and sensitive to mild acidification, differently from TRPM4 (Liman, 2014). This rise and oscillatory waves of intracellular calcium could be induced by the binding of ATP with purinergic receptor on the surface of CCs membrane (Fioretti et al., 2005; Gao et al., 2023), activating a signaling cascade which opens TRPM5. In mouse COC, it was observed that the activation of P2Y2 subtype purinergic receptor elicited a Ca^2+^‐dependent chloride current (*I*_Cl(Ca)_), and a nonselective cation current intracellular Ca^2+^‐independent (Arellano et al., 2002), both different from those which were recorded in our experiments. Recently, the presence of the type 2 taste bitter receptors (TAS2Rs) was identified on both cumulus and GCs (Semplici et al., 2021), and their involvement in steroidogenesis was proposed (Luongo et al., 2022; Luongo et al., 2025). This discovery, coupled with our findings regarding TRPM5 in human CCs, may also open new perspectives on the role of bitter molecules in the reproductive system.

The study of CCs should be carried out on the COC—which represents a limitation of this research—in order to give more valuable insights into the synergistic activity in female physiology, of course taking always into account the ethical issues that may derive. Further studies will be required to better understand the precise mechanism which lies beyond this signaling pathway, as well as the role of other metabolites or specific modulators of the ion channels described above.

To conclude, our study described for the first time the electrophysiological sub‐populations in human CCs. The rise of intracellular calcium induced by 100 μM ATP was found to activate a TRPM5‐like current, and a putative model of cell signaling was proposed. Furthermore, mild acidification may provoke membrane depolarization through the reversible inhibition of K_v_1.5 and TRPM5‐like channels. These results could pave the way for new therapeutic approaches to infertility by targeting key specific ion channels and their regulatory mechanisms with specific blockers or modulators which may help the successful rate of assisted reproductive technologies.

## AUTHOR CONTRIBUTIONS

A.B., R.G., B.F. conceived and designed research. A.B., R.G. analyzed data. A.B., R.G., C.C., M.M. performed experiments. A.B., R.G., B.F. interpreted results of experiments. A.B., R.G., B.F. prepared figures. A.B., R.G., B.F. drafted manuscript. A.B., R.G., B.F., C.C., M.M., A.F., S.G. edited and revised the manuscript. All authors read and approved the final version of the manuscript.

## FUNDING INFORMATION

This research was funded by Italian Ministry of Enterprises and Made in Italy (MIMIT), grant number F/350076/01/X60‐VAPORE‐CUP B99J24001000005, and by Italian Ministry of University and Reasearch (MUR) for financial support by the PRIN, “Progetti di Rilevante Interesse Nazionale” 2022 Fioretti (Role of ovarian sensory excitability in the physiopathology of folliculogenesis. Prot. 202294JF88 to B.F.). The research is also part of a PNRR (Piano Nazionale di Ripresa e Resilienza) scholarship entitled “Shelf‐life enhancer”, supported by the Italian Ministry for Universities and Research (n° 38–0333‐23‐DOT1323112‐2293) to A.B and B.F.

## CONFLICT OF INTEREST STATEMENT

The authors declare no conflicts of interest.

## Supporting information


**Figure S1.** CC‐type 1 current is a voltage‐dependent potassium current. (a) Insensitivity of potassium currents before (CTRL, black trace) and after (red traces) application of 2 mM octanol (three independent experiments). (b) Insensitivity of potassium currents before (CTRL, black trace) and after (red traces) application of 100 μM NPPB (three independent experiments). The protocol included pre‐conditioning the cell at −80 mV for 500 ms, then bringing it to +140 mV from a V holding potential (−60 mV).
**Figure S2.** Nonselective cationic channels is not calcium‐permeable. FURA‐2 calcium imaging performed by applying modified Ringer solution with 1 mM Ca^2+^ and 5 mM Ca^2+^ did not show changes in the intracellular calcium of cumulus cells, suggesting a non‐permeation to the ion (three independent experiments).
**Figure S3.** Immunocytochemistry of cumulus cells sub‐populations. (a) Fluorescence imaging by immunocytochemistry revealed the expression and localization of K_V_1.5 channels in cumulus cells (three independent experiments). K_V_1.5 channel expression was obtained by using primary rabbit IgG polyclonal antibody FITC‐conjugated (1:100). Nuclei were stained with DAPI (1:1000). Brightfield shows the contours of the cell. Scale bar represents 20 μm. (b) Fluorescence imaging by immunocytochemistry revealed the expression and localization of BK_Ca_ channels in cumulus cells (three independent experiments). BK_Ca_ channel expression was obtained by using primary rabbit IgG polyclonal antibody (1:100) and Alexa Fluor 555‐conjugated goat anti‐rabbit (1:500). Nuclei were stained with DAPI (1:1000). Brightfield shows the contours of the cell. Scale bar represents 20 μm. (C) Fluorescence imaging by immunocytochemistry revealed the expression and localization of TRPM5 channels in cumulus cells (three independent experiments). TRPM5 channel expression was obtained by using primary rabbit IgG antibody (1:100) and Alexa Fluor 555‐conjugated goat anti‐rabbit (1:500). Nuclei were stained with DAPI (1:1000). Brightfield shows the contours of the cell. Scale bar represents 20 μm.
**Figure S4.** Transcriptomic analysis of cumulus cell sub‐populations. *KCNA5*, *TRPM5* and *KCNMA1* gene expression was performed by rt‐PCR to confirm the molecular nature of the ion channels. Empty dots represent the relative gene expression of each patient, whereas the black dot is the mean ± SE (*n* = 7, from seven different patients).

## Data Availability

The original contributions presented in the study are included in the article. Further inquiries can be directed at the corresponding authors.
