# Supplementary material for: Extracellular acidity and ATP modulate ion currents in human cumulus cells indicating possible roles as metabolic sensors of the follicular microenvironment
Source: Physiol Rep. 2026 Jan 28;14(2):e70729. doi: 10.14814/phy2.70729 (PMC12849215; doi:10.14814/phy2.70729)
Supplement: Supplementary file 1 — Figure S1. CC‐type 1 current is a voltage‐dependent potassium current. (a) Insensitivity of potassium currents before (CTRL, black trace) and after (red traces) application of 2 mM octanol (three independent experiments). (b) Insensitivity of potassium currents before (CTRL, black trace) and after (red traces) application of 100 μM NPPB (three independent experiments). The protocol included pre‐conditioning the cell at −80 mV for 500 ms, then bringing it to +140 mV from a V holding potential (−60 mV). Figure S2. Nonselective cationic channels is not calcium‐permeable. FURA‐2 calcium imaging performed by applying modified Ringer solution with 1 mM Ca2+ and 5 mM Ca2+ did not show changes in the intracellular calcium of cumulus cells, suggesting a non‐permeation to the ion (three independent experiments). Figure S3. Immunocytochemistry of cumulus cells sub‐populations. (a) Fluorescence imaging by immunocytochemistry revealed the expression and localization of KV1.5 channels in cumulus cells (three independent experiments). KV1.5 channel expression was obtained by using primary rabbit IgG polyclonal antibody FITC‐conjugated (1:100). Nuclei were stained with DAPI (1:1000). Brightfield shows the contours of the cell. Scale bar represents 20 μm. (b) Fluorescence imaging by immunocytochemistry revealed the expression and localization of BKCa channels in cumulus cells (three independent experiments). BKCa channel expression was obtained by using primary rabbit IgG polyclonal antibody (1:100) and Alexa Fluor 555‐conjugated goat anti‐rabbit (1:500). Nuclei were stained with DAPI (1:1000). Brightfield shows the contours of the cell. Scale bar represents 20 μm. (C) Fluorescence imaging by immunocytochemistry revealed the expression and localization of TRPM5 channels in cumulus cells (three independent experiments). TRPM5 channel expression was obtained by using primary rabbit IgG antibody (1:100) and Alexa Fluor 555‐conjugated goat anti‐rabbit (1:500). Nuclei were stained with D [file PHY2-14-e70729-s001.docx]

**SUPPLEMENTARY FILE**

**
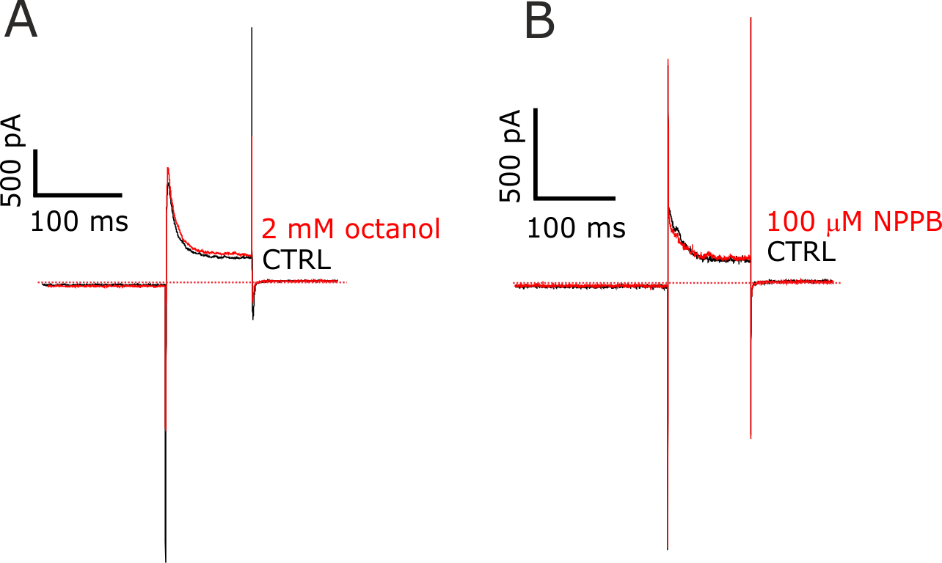
**

**Figure S1. CC-type 1 current is a voltage-dependent potassium current. A)** Insensitivity of potassium currents before (CTRL, black trace) and after (red traces) application of 2 mM octanol (three independent experiments). **B)** Insensitivity of potassium currents before (CTRL, black trace) and after (red traces) application of 100 μM NPPB (three independent experiments). The protocol included pre-conditioning the cell at −80 mV for 500 ms, then bringing it to +140 mV from a V holding potential (−60 mV).

**
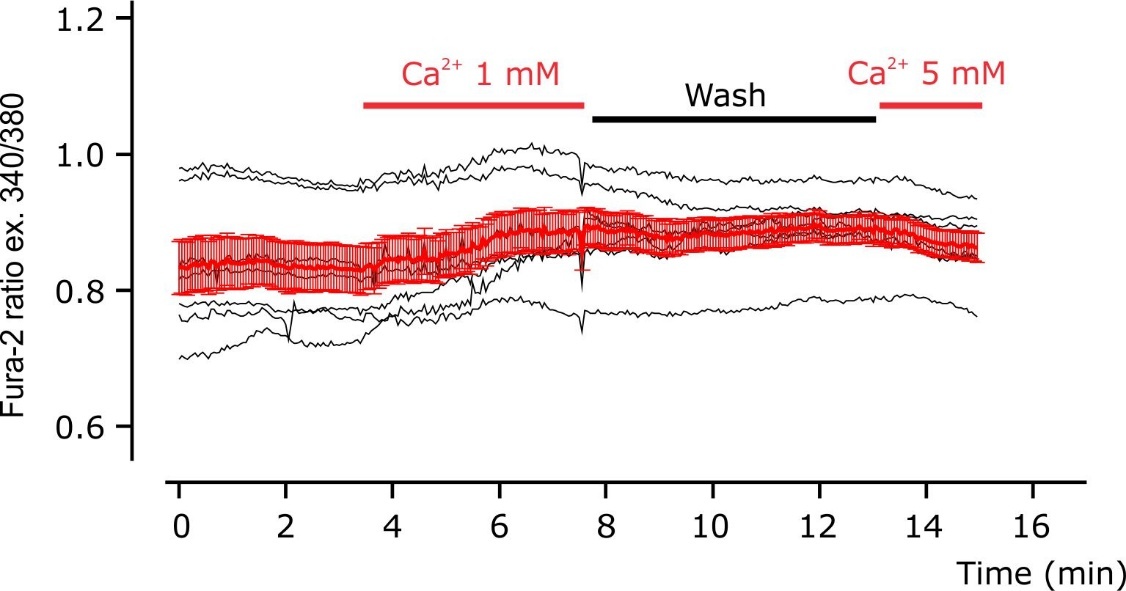
**

**Figure S2. Nonselective cationic channels is not calcium-permeable.** FURA-2 calcium imaging performed by applying modified Ringer solution with 1 mM Ca^2+^ and 5 mM Ca^2+^ did not show changes in the intracellular calcium of cumulus cells, suggesting a non-permeation to the ion (three independent experiments).

**
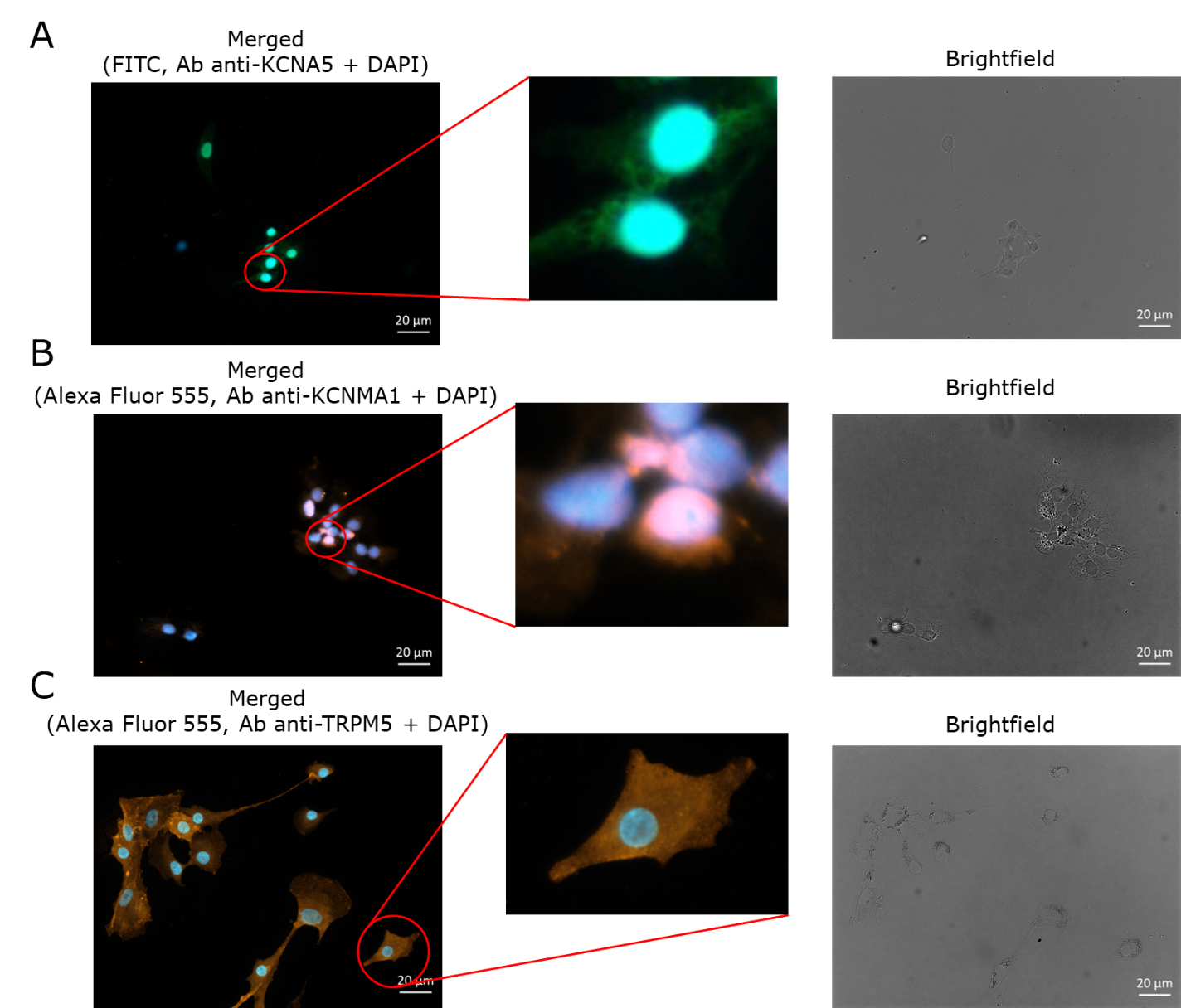
Figure S3. Immunocytochemistry of cumulus cells sub-populations. A)** Fluorescence imaging by immunocytochemistry revealed the expression and localization of K_V_1.5 channels in cumulus cells (three independent experiments). K_V_1.5 channel expression was obtained by using primary rabbit IgG polyclonal antibody FITC-conjugated (1:100). Nuclei were stained with DAPI (1:1000). Brightfield shows the contours of the cell. Scale bar represents 20 μm (magnification 40x). **B)** Fluorescence imaging by immunocytochemistry revealed the expression and localization of BK_Ca_ channels in cumulus cells (three independent experiments). BK_Ca_ channel expression was obtained by using primary rabbit IgG polyclonal antibody (1:100) and Alexa Fluor 555-conjugated goat anti-rabbit (1:500). Nuclei were stained with DAPI (1:1000). Brightfield shows the contours of the cell. Scale bar represents 20 μm (magnification 40x). **C)** Fluorescence imaging by immunocytochemistry revealed the expression and localization of TRPM5 channels in cumulus cells (three independent experiments). TRPM5 channel expression was obtained by using primary rabbit IgG antibody (1:100) and Alexa Fluor 555-conjugated goat anti-rabbit (1:500). Nuclei were stained with DAPI (1:1000). Brightfield shows the contours of the cell. Scale bar represents 20 μm (magnification 40x).

**
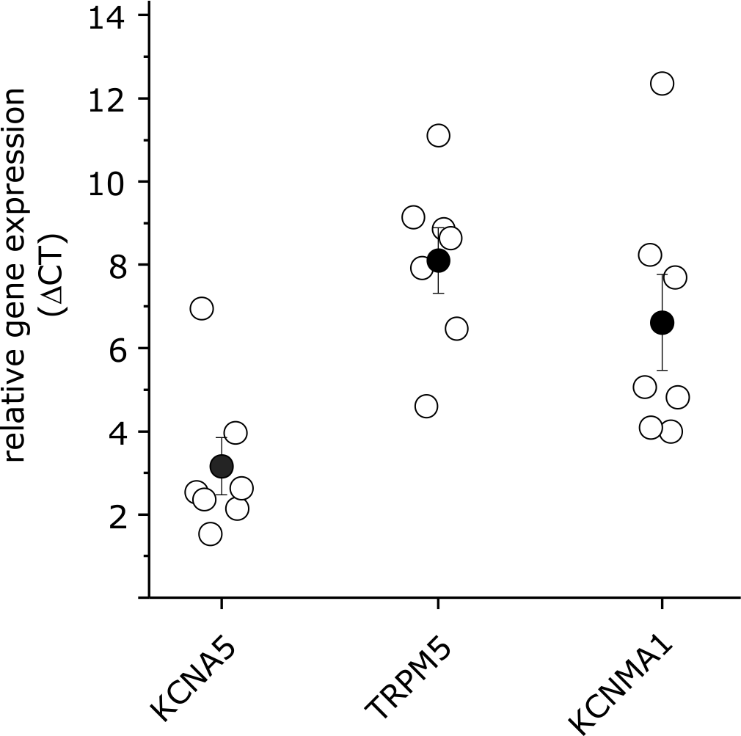
**

**Figure S4. Transcriptomic analysis of cumulus cell sub-populations.** *KCNA5*, *TRPM5* and *KCNMA1* gene expression was performed by rt-PCR to confirm the molecular nature of the ion channels. Empty dots represent the relative gene expression of each patient, whereas the black dot is the mean ± SE (n=7, from seven different patients).
